# Integrated miRNAome and Transcriptome Analysis Reveals Argonaute 2-Mediated Defense Responses Against the Devastating Phytopathogen *Sclerotinia sclerotiorum*

**DOI:** 10.3389/fpls.2020.00500

**Published:** 2020-04-29

**Authors:** Jia-Yi Cao, You-Ping Xu, Xin-Zhong Cai

**Affiliations:** ^1^Zhejiang Provincial Key Laboratory of Crop Pathogen and Insect Biology, Institute of Biotechnology, College of Agriculture and Biotechnology, Zhejiang University, Hangzhou, China; ^2^Key Laboratory of Applied Marine Biotechnology, Ningbo University, Ministry of Education of China, Ningbo, China; ^3^Centre of Analysis and Measurement, Zhejiang University, Hangzhou, China

**Keywords:** AGO2, defense, miRNAome, *Sclerotinia sclerotiorum*, transcriptome

## Abstract

Argonaute 2 (AGO2)-mediated role in plant defense against fungal pathogens remains largely unknown. In this study, integrated miRNAome and transcriptome analysis employing *ago2* mutant was performed to reveal AGO2-associated miRNAs and defense responses against the devastating necrotrophic phytopathogen *Sclerotinia sclerotiorum*. Both miRNAome and transcriptomes of *S. sclerotiorum*-inoculated *ago2-1* mutant (*ago2-Ss*) and wild-type (WT*-Ss*) as well as mock-inoculated *ago2-1* mutant (*ago2*) and wild-type (WT) Arabidopsis plants, were analyzed by sRNA and mRNA deep sequencing. Differentially expressed genes (DEGs) and differentially expressed miRNAs (DEMs) of the comparisons WT*-Ss*/WT, *ago2*/WT, *ago2-Ss*/WT*-Ss*, and *ago2-Ss*/*ago2* were identified. Furthermore, integration analysis for the DEMs and DEGs identified over 40 potential AGO2-dependent *Sclerotinia sclerotiorum*-responsive (ATSR) DEM-DEG pairs involving modulation of immune recognition, calcium flux, redox homeostasis, hormone accumulation and signaling, cell wall modification and metal ion homeostasis. Data-mining result indicated that most of the DEMs were bound with AGO2. Moreover, Arabidopsis mutant analysis demonstrated that three ROS and redox homeostatasis related DEGs of identified DEM-DEG pairs, *GSTU2*, *GSTU5*, and *RBOHF* contributed to the AGO2-mediated defense against *S. sclerotiorum*. This work provides genome-wide prediction of miRNA–target gene pairs that are potentially associated with the AGO2-dependent resistance against *S. sclerotiorum*.

## Introduction

*Sclerotinia sclerotiorum*, causing white mold/stem rot disease in oil and vegetable crops, is one of the most devastating necrotrophic fungal pathogens (Bolton et al., 2006). Plant immunity toward *S. sclerotiorum* appears to be under complex genetic control. Phytohormones such as ET and JA, NO, ROS, and autophagy are important regulators of defense against *S. sclerotiorum* (Guo and Stotz, 2007; Perchepied et al., 2010; Williams et al., 2011; Kabbage et al., 2013). Some *MAPK* and *WRKY* genes are also involved in resistance against this pathogen (Wang et al., 2009, 2014). In addition, *S. sclerotiorum* PAMP triggered immunity (PTI) in *Arabidopsis thaliana* has been identified (Zhang et al., 2013). Our previous studies revealed that proteins involved in the calcium-triggered pathway such as CNGC, CaM, CDPK, CRK, and CCaMK contribute to the regulation of plant resistance to *S. sclerotiorum* (Saand et al., 2015a, b; Wang et al., 2015; Rahman et al., 2016; Wang J.-P. et al., 2016). Nevertheless, mechanism underlying plant resistance against this pathogen is still far from fully understood.

Recently, we found that oilseed rape-*S. sclerotiorum* interactions are under tight control by miRNAs, and AGOs-mediated RNA silencing plays an important role in resistance to *S. sclerotiorum* (Cao et al., 2016b). AGO2 is one of the most important plant AGOs. It contributes greatly to antiviral silencing independently or in cooperation with other AGOs by either binding to vsiRNAs to slice target viral RNAs (Takeda et al., 2008; Harvey et al., 2011; Jaubert et al., 2011; Scholthof et al., 2011; Wang et al., 2011; Carbonell et al., 2012; Zhang et al., 2012; Garcia-Ruiz et al., 2015; Ma et al., 2015) or binding to vasiRNAs to silence plant genes related to antiviral defense regulation (Carbonell and Carrington, 2015; Fatyol et al., 2016). AGO2 is also involved in resistance to the bacterial pathogen *Pseudomonas syringae* pv. *tomato* by binding miR393b^∗^ to silence its target gene *MEMB12* thereby promoting the exocytosis of antimicrobial PR proteins (Zhang et al., 2011). In addition, AGO2 plays a role in DSB repair by binding to diRNAs (Wei et al., 2012). Despite the essential role of AGO2 in antiviral and antibacterial defenses, its role in antifungal defense remains obscure. We previously reported that two miRNAs miR403 and miR403a-3p, which target *AGO2*, are down-regulated in response to *S. sclerotiorum.* Moreover, *A. thaliana ago2-1* mutant plants were more susceptible than the WT plants to *S. sclerotiorum* (Cao et al., 2016b). This result supports a role of AGO2 in defense response to *S. sclerotiorum* and prompts us to further probe the mechanisms underlying the AGO2-dependent defense against the devastating necrotrophic fungal pathogen *S. sclerotiorum.*

In this study, correlative analyses for both miRNAome and transcriptomes of four types of materials, *S. sclerotiorum*-inoculated *ago2-1* mutant (*ago2-Ss*) and WT*-Ss* as well as mock-inoculated *ago2-1* mutant (*ago2*) and WT Arabidopsis plants, were performed to identify AGO2-associated miRNAs and defense responses against *S. sclerotiorum* at the genome-wide level, thereby establishing a link of AGO2-dependent RNA silencing to tolerance against the necrotrophic fungal pathogen.

## Materials and Methods

### Plant Materials and Pathogen Inoculation Procedure

Arabidopsis plants of the ecotype Columbia 0 (Col-0) and mutants in Col-0 backgroud *ago2-1* (SALK_003380, Lobbes et al., 2006), *gstu2-1* (SALK_051158C), *gstu2-2* (SALK_062920C), *gstu5-1* (SALK_107148C), *gstu5-2* (CS855195), and *rbohf* (Torres et al., 2002) were grown in growth cabinets at 23°C and 70% RH with a 12/12 h day/night photoperiod. *S. sclerotiorum* mycelial plugs were prepared as described (Cao et al., 2016b) and were used to inoculate Arabidopsis leaves. One fully developed leaf per plant was inoculated with four mycelial plugs of 3 mm in diameter. As a mock-inoculation control, leaves were inoculated with empty PDA plugs. The inoculated plants were grown under conditions described above except that the RH was increased to 90%. The mock treated (WT-mock, *ago2-1*-mock) and *S. sclerotiorum*-inoculated (WT-*Ss*, *ago2-1-Ss*) leaves were collected at 8 h post-inoculation (hpi), immediately frozen in liquid nitrogen and kept at −80°C until use. Three independent plants were pooled to yield one biological replicate and each treatment consisted of three independent biological replicates.

### Transcriptome Library Construction, Sequencing, and Annotation

The total RNA was extracted using Trizol reagent (Invitrogen, Carlsbad, United States) and purified using RNeasy MiniElute Cleanup Kit (Qiagen, Hilden, Germany) according to the manufacturer’s protocol. The RNA’s purity and concentration were determined using a Qubit RNA Assay Kit and the RNA sample integrity was verified by the RIN (RNA integrity number) value that was determined using Agilent 2100 Bioanalyzer (Santa Clara, CA, United States). Samples with RIN of more than 7 were used for further analysis. The poly-A containing mRNA molecules were purified from 1 μg of total RNA by using poly-T oligo-attached magnetic beads. The purified mRNA was cleaved into short fragments (about 200 nt) and was then reversely transcribed into first strand cDNA using random hexamers, following by second strand cDNA synthesis using DNA polymerase I and RNase H. The cDNA fragments were purified, end blunted, “A” tailed, and adaptor ligated. PCR was used to selectively enrich those DNA fragments that have adapter molecules on both ends and to amplify the amount of DNA in the library. The number of PCR cycles was minimized to avoid skewing the representation of the library. The libraries were qualified by 2100 Bioanalyzer chip and quantified by KAPA SYBR rapid quantitative PCR Kit (KAPA Biosystem, Wilmington, United States, Cat no.KK4602). The produced libraries were sequenced on the HiSeq 2500 platform by CapitalBio Corporation (Beijing, China).

The raw image data was transformed by base-calling into sequence data and stored in FASTQ format to get the raw reads. A NGSQC software (Patel and Jain, 2012) was used to remove adaptor sequences and low-quality sequences from the raw reads. The retained high-quality pair-end reads of Arabidopsis for each sample were mapped to the Arabidopsis genome sequences (version TAIR10.29) by a HISAT software (Kim et al., 2015). The number of mapped clean reads for each gene was then counted and normalized into the FPKM by Cuffquant and Cuffnorm softwares (Trapnell et al., 2012). Cuffdiff software (Trapnell et al., 2013) was used to identify DEGs. Expression difference with a ratio of ≥2.0 and ≤0.5 and a *q*-Value (FDR-adjusted *p*-Value) of ≤0.05 was considered as significant up-regulation and down-regulation, respectively.

Gene ontologies and KEGG pathways were assigned to each unigenes using Blast2GO (Conesa et al., 2005). Gene ontology (GO) enrichment analysis was performed using online tools for DAVID bioinformatics resources^1^ (Huang et al., 2009).

### Small RNA Sequencing and Analysis

Small RNA sequencing libraries were generated using NEBNext Multiplex Small RNA Library Prep Set for Illumina (NEB, MA, United States) following manufacturer’s instructions. Briefly, NEB 3′ SR adaptor was ligated to 3′ end of small RNAs in the total RNA extracts. The SR RT Primer was hybridized to 3′ SR Adaptor to prevent adaptor-dimer formation. The 5′ SR adaptor was then ligated to 5′ end of small RNAs. Then first strand cDNA was synthesized using M-MuLV reverse transcriptase (RNase H^–^). PCR amplification was performed using Long Amp Taq 2X Master Mix, SR Primer for illumina and index (X) primer. PCR products were purified on a 8% polyacrylamide gel and DNA fragments of 145∼160 bp (the length of small non-coding RNA plus the 3′ and 5′ adaptors) were recovered. Library quality was assessed on the Agilent Bioanalyzer 2100 system using DNA High Sensitivity Chips. The librariy preparations were sequenced on an Illumina Hiseq 2500 platform at the CapitalBio Corporation (Beijing, China) following the vendor’s recommended protocol.

Adaptor and low-quality sequences were removed, and all identical sequences were counted and eliminated from the initial data set. The unique reads were mapped to the Arabidopsis genome (version TAIR10.29) using the program Bowtie (Langmead et al., 2009). Sequences that matched Arabidopsis rRNA, scRNA, snoRNA, snRNA, or tRNA sequences in the NCBI^2^ and Rfam RNA family databases were filtered out (Gardner et al., 2009; Barrett et al., 2013). In addition, sequences shorter than 17 nt or longer than 35 nt and those overlapping exons and introns in the mRNAs, were also removed. Sequences that perfectly matched miRNA precursors and mature miRNAs in the Sanger miRBase^3^ of Arabidopsis were identified as known miRNAs. The sequences that matched miRBase entries of other plant species, but not Arabidopsis, were designated as conserved miRNAs. To identify potentially novel miRNAs, the software Mireap^4^ was used to predict precursor sequences and their secondary structures.

The expression of miRNAs was normalized and those with a *p*-Value of ≤0.05 and fold changes of ≥1.5 or ≤0.67 were identified as significantly differentially expressed between the two samples.

All the raw data obtained from transcriptome and miRNAome sequencing analyses have been deposited to the NCBI SRA database under the accession numbers PRJNA607858 and PRJNA607657, respectively.

### Integrated Analysis of mRNA-seq and miRNA-seq

In order to define the likely functional miRNA-mRNA pairs, the software PITA with energy ≤−10 kcal/mol (Kertesz et al., 2007) and miRanda with score ≥140 and with energy ≤−10 kcal/mol (John et al., 2005) were used to predict potential gene targets for the identified DEMs. The top 10 targets predicted by software PITA or miRanda and the overlap targets predicted by both softwares were collected as the potential targets of the analyzed DEMs. The predicted target genes of the DEMs were then searched against the corresponding DEG pools to obtain the DEGs that were predicted to be the target of DEMs. These selected DEM-DEG pairs were subjected for comparison of expression tendency between the corresponding DEGs and DEMs of the pairs, and the DEM-DEG pairs with reverse expression correlation relationship were collected to construct the miRNA-mRNA regulatory network.

### Expression Analyses of mRNA and miRNA

Quantitative reverse transcription-PCR (qRT-PCR) was conducted to analyze the expression of DEMs and mRNAs. To check the expression of miRNAs, we used the Mir-X^TM^ miRNA first-strand synthesis kit (Clontech) for reverse transcription reaction and the primers were designed for the 6 selected miRNAs using U6 snRNA as an internal control (Supplementary Table S1). Primers for qRT-PCR for 5 DEGs as well as their reference gene which encodes a 18S ribosomal protein in Arabidopsis, were also listed in Supplementary Table S1. The qRT-PCR analyses were conducted three times, each containing three replicates for all genes. Relative gene expression was quantified by 2^–ΔΔCt^ calculation (Cao et al., 2016b). The statistical data analyses were conducted as described (Cao et al., 2016b).

## Results

### Transcriptome for AGO2-Mediated Defense Responses to *S. sclerotiorum*

In order to understand *AGO2*-mediated and/or *S. sclerotiorum*-induced responses, the genes differentially expressed in between Arabidopsis plants of the WT and *ago2-1* mutant under normal condition or in response to *S. sclerotiorum*, were identified by performing RNA-seq analysis for mock- and *S. sclerotiorum*-inoculated (8 hpi) *ago2-1* and WT plants. Twelve cDNA libraries from samples of three independent biological replicates were constructed with total RNA and subjected to Illumina deep sequencing analyses. An overview of the sequencing and assembly data is shown in Supplementary Table S2. Four sets of gene expression comparisons were undertaken to obtain genes involved in: (1) *S. sclerotiorum*-induced responses; (2) *AGO2*-mediated responses; and (3) *S. sclerotiorum*-induced and *AGO2*-mediated responses, respectively.

**FIGURE 1:**
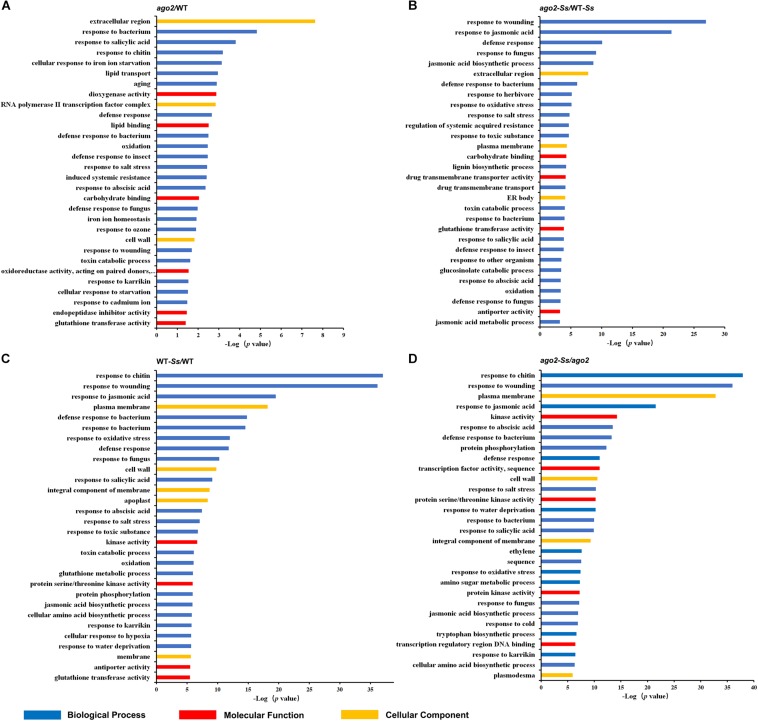
Top 30 significantly enriched GO terms for differentially expressed genes for comparisons between *ago2-1* mutant and wild-type (WT) plants (*ago2*/WT, **A**), *S. sclerotiorum*-inoculated *ago2-1* mutant and WT plants (*ago2-Ss*/WT-*Ss*, **B**), *S. sclerotiorum*-inoculated and mock-inoculated WT plants (WT-*Ss*/WT, **C**) and *S. sclerotiorum*-inoculated and mock-inoculated *ago2-1* mutant plants (*ago2-Ss*/*ago2*, **D**).

To identify Arabidopsis genes modulated in response to *S. sclerotiorum*, those whose expression was significantly altered by two folds or more in three replicates with a *q-*Value ≤ 0.05 between *S. sclerotiorum*- and mock-infected WT plants (WT-*Ss* vs WT) were collected. There were a total of 2223 this type of DEGs (Supplementary Figure S1 and Supplementary Table S3A). The KEGG pathway analysis demonstrated that these genes belonged to 23 pathways, including two defense-related pathways, “plant-pathogen interaction” and “glucosinolate biosynthesis” and one hormone-related pathway (“plant hormone signal transduction”) (Supplementary Table S4A). GO terms were enriched with those related with defense response and signaling (Figure 1C and Supplementary Table S6A). The DEGs which were involved in defense response, hormone signaling or redox homeostasis were listed in Supplementary Table S5. Interestingly, *PAD3*, a key gene in camalexin synthesis pathway and defense against *S. sclerotiorum* (Stotz et al., 2011), was most up-regulated (by 1136.5 folds) after *S. sclerotiorum* inoculation (Supplementary Table S5), indicating that some of these DEGs identified in this study might be involved in defense against *S. sclerotiorum*. Besides, sets of resistance genes, defensin-like, cell-wall related genes, MAPK signaling pathway genes and a series of PTI and DAMP triggered immunity (DTI) related genes were also in the group of defense response DEGs (Supplementary Table S5). DEGs belonging to the hormone signaling group were mainly involved in ET and JA pathways, with several related to auxin and ABA and one to SA (*SAR Deficient 1*). Redox homeostasis related DEGs included *SOD*, *RbohD*, *Trx* and some members of *GST* gene family. The remaining DEGs in the comparison of WT-*Ss*/WT, including *LTP*, *CNGC* and *WRKY33*, were listed in Supplementary Table S3A. Collectively, these genes may be involved in the interaction between *S. sclerotiorum* and Arabidopsis and were the important candidates to reveal the Arabidopsis immunity mechanism toward *S. sclerotiorum*.

To identify potential AGO2 targeted genes, mRNA transcriptomes of *ago2-1* mutant and WT plants in the absence of pathogen were compared (*ago2*/WT), which resulted in identification of 119 DEGs (Supplementary Figure S1 and Supplementary Tables S3B, S4B). The GO enrichment analyses showed that 31 biological process GO terms for these DEGs were significantly enriched (*p*-Value ≤ 0.05) (Supplementary Table S6B). Interestingly, majority of them were related with defense response and resistance (Figure 1A). DEGs of this comparison group also contained some important genes related to defense response, hormone signaling or redox homeostasis, including genes encoding defensins, thionin-2.2, basic endochitinase B, jacalin-related lectin 22, NIMIN1, ERF, and GSTs (Supplementary Table S7). These results suggested that AGO2 may constitutively affect the resistance of Arabidopsis to *S. sclerotiorum* by regulating these defense-related genes or pathways.

To further identify genes that are both affected by AGO2 and involved in susceptibility to or defense against *S. sclerotiorum*, mRNA transcriptomes of *S. sclerotiorum*-infected *ago2-1* mutant and WT plants (*ago2-Ss*/WT-*Ss*) were compared. A total of 759 DEGs of this comparison were obtained (Supplementary Figure S1 and Supplementary Table S3C). The KEGG pathway analysis indicated that these DEGs were enriched in the pathway “ath04075:Plant hormone signal transduction” and some pathways related with defense-associated metabolisms (Supplementary Table S4C). The GO enrichment analyses indicated that the top 30 of GO terms were enriched with defense-related terms such as “response to jasmonic acid,” “defense response,” “response to fungus,” “jasmonic acid biosynthetic process,” and “regulation of systemic acquired resistance” (Figure 1B and Supplementary Table S6C). Strikingly, three of the top 30 GO terms including two of the top 5 were related with jasmonic acid, including “response to jasmonic acid” (top 2), “jasmonic acid biosynthetic process” (top 5), and “jasmonic acid metabolic process” (Figure 1B). Moreover, the first-ranked “response to wounding” is well-known to be related with jasmonic acid pathway. Together, these data indicated that AGO2-mediated defense against *S. sclerotiorum* heavily involves jasmonic acid pathway. To analyze AGO2-mediated defense in detail, the DEGs belonged to this comparison group and related to defense response, hormone signaling or redox homeostasis were listed in Supplementary Table S8. Among them were genes encoding NBS-LRR class and MOL-like disease-resistant proteins, PEPR2, Defensin-like, DIRs, JRLs, MPKKKs, TIFYs, BAP2, EDS5, NIMIN1, ECS1, a set of proteins related to ET, JA, and auxin pathways and a set of proteins related with redox homeostasis, including Copper chaperone for SOD, RbohC, RbohF, GSTs and peroxidases (Supplementary Table S8). JA related DEGs included genes encoding allene oxide synthase, 12-oxophytodienoate reductase 3, jasmonate O-methyltransferase and MYC2 (Supplementary Table S8). Other interesting DEGs not listed in Supplementary Table S8 included *CDPK29*, *nsLTP3*, *WRKY48*, *WAK3*, *Germin-like protein subfamily T member 2* (Supplementary Table S3C). Collectively, JA pathway and other genes listed above may be involved in the AGO2-mediated resistance to *S. sclerotiorum*.

Differentially expressed genes of the comparison *ago2-Ss*/WT-*Ss* are candidate *Ss*-responsive genes affected by but may be not directly regulated by AGO2. To identify candidate AGO2-dependent *Ss*-responsive (ATSR) genes, we further selected out the DEGs that were expressed significantly differentially in both WT-*Ss*/WT and *ago2-Ss*/*ago2* comparisons but to the opposite direction (Figures 1C,D and Supplementary Tables S3A,D, S4A,D, S6A,D). Consequently, seven candidate ATSR genes were found out. They include six protein-coding genes that encode for a MD-2-related lipid recognition domain-containing protein, Fe superoxide dismutase 1 (FSD1), Nitrilase 2 (NIT2), an alpha/beta-hydrolases superfamily protein, a TRAF-like family protein and FEP2 (Fe-uptake-inducing peptide 2)/IMA2 (IRONMAN 2) and one non-coding gene, a MIR169 gene (Table 1). Most of the ATSR genes were reported to be related with defense regulation (see references in Table 1). Deciphering the functions of these ATSR genes should provide insights into the mechanisms underlying AGO2-mediated resistance against *S. sclerotiorum*. Additionally, 1897 genes were only significantly differentially expressed in either WT-*Ss*/WT or *ago2-Ss*/*ago2* (Supplementary Table S9A), and 1754 genes were significantly differentially expressed in both comparisons in the same direction (both up-regulated or down-regulated) (Supplementary Table S9B). These genes might be less strongly, partially or indirectly modulated by AGO2 and/or less tightly, partially or indirectly involved in the interactions between Arabidopsis and *Ss*.

**TABLE 1:** Potential defense-related ATSR genes.

Target gene ID	Fold Change (*q*-Value)		Description	Function
	WT-*Ss*/WT	*ago2-Ss*/*ago2*		
AT5G23820	0.31 (0)	2.08 (0)	MD-2-related lipid recognition domain-containing protein	Immune recognition
AT4G25100	0.34 (0)	3.43 (0)	FSD1 (Superoxide dismutase [Fe] 1)	ROS scavenger
AT4G17470	0.28 (0)	3.75 (0)	Alpha/beta-Hydrolases superfamily protein	Unknown function
AT3G26815| AT3G26816	3.85 (0)	0.28 (0)	MIR169K/L	Disease resistance
AT3G44300	NA (0)	0.37 (0)	NIT2 (Nitrilase 2)	Disease resistance
AT3G28220	0.42 (0)	2.20 (0)	TRAF-like family protein	Immune receptor turnover
AT1G47395	2.30 (0.01)	0.37 (0.01)	FEP2 (Fe-uptake-inducing peptide 2)/IMA2 (IRONMAN 2)	Fe transporter

**FIGURE 2:**
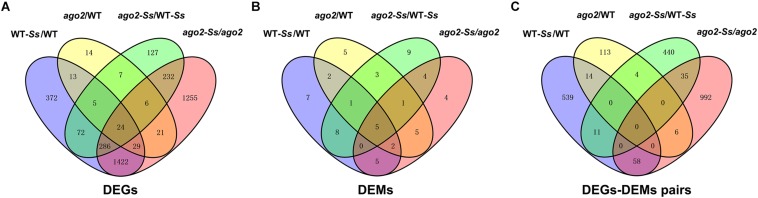
The Venn diagrams to demonstrate the extent of overlapping of DEGs **(A)**, DEMs **(B)** and DEM-DEG pairs **(C)** from various comparisons.

Furthermore, to understand the relationship between the DEGs of various comparisons, a Venn diagram to show the extent of overlapping was constructed (Figure 2A). It showed that 24 DEGs were differentially expressed in all four comparisons. To our interest, among the 759 DEGs of the comparison *ago2-Ss*/WT-*Ss*, 632 shared with those of the comparisons WT-*Ss*/WT and *ago2*/WT, while the remaining 127 were differentially expressed exclusively in *ago2-Ss*/WT-*Ss*, implying that expression of these 127 DEGs is not constitutively AGO2-dependent rather only affected by AGO2 in response to *S. sclerotiorum*. Among them were genes encoding disease resistant proteins WRKYs various proteins related to defense hormone pathways and numerous proteins related with redox homeostasis, such as GSTs (Supplementary Table S10).

### MicroRNAome for AGO2-Mediated Defense Responses to *S. sclerotiorum*

To capture the miRNA expression profiles of Arabidopsis plants of the WT and *ago2-1* mutant constitutively and after inoculation with *S. sclerotiorum*, the same set of libraries for above-mentioned RNA-seq were used for sRNA deep sequencing analyses. An overview of reads for sRNA-seq is shown in Supplementary Table S11. When aligned with the mature Arabidopsis miRNAs, 198 known mature miRNAs deposited in miRBase 21.0 were obtained from the 12 sRNA libraries (Supplementary Table S12). Besides, 31 potentially novel Arabidopsis miRNAs, which were not deposited in miRBase 21.0, were identified (Supplementary Table S12). After normalization of the raw sequence read numbers, the average normalized reads of three independent biological replicates were then used for further comparative analysis of miRNA expression level. We used stringent criteria (the average value of three independent biological replicates conformed to the parameters: *p-*Value ≤ 0.05, fold change ≥1.5) to identify miRNAs that were differentially expressed. The same sets of comparisons as for mRNA transcriptome analyses were undertaken to obtain miRNA genes involved in the three types of responses as described for mRNA transcriptome analyses.

In the comparison between *S. sclerotiorum*-inoculated and mock-inoculated WT plants (WT-Ss/WT), 30 miRNAs were differently expressed, including 22 known and 8 potential novel miRNAs (Supplementary Table S12). Among these 30 DEMs, six exhibited reads of higher than 100, including ath-miR398b-3p/ath-miR398c-3p, ath-miR8175, ath-miR5642a, 1_41269-5p and 5_31160-3p. Ath-miR398b-3p/ath-miR398c-3p had the highest reads (normalized reads 676) among known miRNAs while 1_41269-5p did (normalized reads 6491) among novel miRNAs. They were down-regulated after *S. sclerotiorum-inoculation* by 2.8 and 1.5 folds, respectively. These results indicated that these DEMs, especially those with high reads, may regulate the resistance of Arabidopsis to *S. sclerotiorum.*

In the comparison group *ago2/*WT, there were 24 DEMs, containing 23 known and 1 potential novel miRNAs (Supplementary Table S12). Six of them had reads of over 100, including ath-miR398b-3p/ath-miR398c-3p, ath-miR393b-3p, ath-miR168a-3p, ath-miR5642a, and 1_41269-5p. These miRNAs were highly likely to be regulated by AGO2 in a direct or indirect manner.

Further comparison between *S. sclerotiorum* infected *ago2-1* and WT plants (*ago2-Ss*/WT-*Ss*) resulted in identification of 32 DEMs including 25 known and 7 potential novel miRNAs (Supplementary Table S12). Five of them, ath-miR398b-3p/ath-miR398c-3p, ath-miR393b-3p, ath-miR8175 and 5_31160-3p, displayed reads of higher than 100. These miRNAs are good candidates associated with AGO2 in response to *S. sclerotiorum* and thus involved in AGO2-mediated miRNA-dependent regulation of the interaction between Arabidopsis and *S. sclerotiorum*.

To further identify the ATSR miRNAs, the DEMs that were expressed significantly differentially in both WT-*Ss*/WT and *ago2-Ss*/*ago2* comparisons but to the opposite direction were screened out. A total of six candidate ATSR miRNAs were obtained. They were ath-miR397b, ath-miR398b-3p, ath-miR398b-5p, ath-miR398c-3p, ath-miR398c-5p, and ath-miR408-3p (Supplementary Table S12). Among them, ath-miR398b-3p and ath-miR398c-3p were also DEMs for *ago2-Ss*/WT-*Ss* comparison and displayed reads of over 100 (Supplementary Table S12), and thus were highly promising ATSR miRNAs.

Moreover, the Venn diagram of DEMs of the four comparisons (Figure 2B) indicated that 5 DEMs were differentially expressed in all four comparisons. Intriguingly, among the 31 DEMs of the comparison *ago2-Ss*/WT-*Ss*, 22 overlapped with those of the comparisons WT-*Ss*/WT, *ago2*/WT and *ago2-Ss/ago2*, while the remaining 9 were differentially expressed only in *ago2-Ss*/WT-*Ss*, suggesting that expression of these 9 DEMs is not constitutively AGO2-dependent rather only affected by AGO2 in response to *S. sclerotiorum*.

### Identification of Reversely Correlated DEM-DEG Pairs Associated With AGO2-Mediated Defense Responses to *S. sclerotiorum*

Given that, in most cases, miRNAs negatively regulate the expression of their target mRNAs by target RNA cleavage, the expression patterns of miRNA target genes generally show an inverse correlation with those of miRNAs. Therefore, for the majority of cases that involve target cleavage, the simple expectation is that when expression of miRNAs is induced, that of their target mRNAs is reduced and vice versa (Jones-Rhoades et al., 2006). Therefore, following the above individual analyses of miRNAs and mRNAs, correlation analysis for the DEMs and DEGs identified above was further performed to identify the differentially expressed miRNA-target mRNA pairs from the DEMs and DEGs, thereby provide some insights into the regulatory mechanisms underlying AGO2-mediated and/or defense against *S. sclerotiorum*.

A total of 622 reversely expressed miRNA-predicted target mRNA pairs involving 29 DEMs and 388 DEGs were identified in the comparison group WT-*Ss*/WT (Supplementary Table S13). Among these pairs, the most interesting ones to us are those related to resistance (Supplementary Table S14). For example, ath-miR398b-3p miRNA was reduced by 2.8 folds after *S. sclerotiorum* inoculation while its predicted target mRNAs NIMIN1, which modulates the expression of *PR* genes through interacting with NPR1 and TGA transcription factors, was induced by 4.2 folds. More interestingly, a novel miRNA 1_41269-5p, with quite high reads (6491), was significantly down-regulated after inoculation (by 2.1 folds). Many of its predicted target genes, showing an inverse correlation with this miRNA, have previously been reported to play an important role in plant defense response, including the most up-regulated (1136.5 folds) defense gene *PAD3*, *WRKY48*, E3 ubiquitin-protein ligase gene *PUB22*, disease resistance genes (TIR-NBS-LRR class), metacaspase 9 gene *MC9*, cyclic nucleotide-gated ion channel gene *CNGC11*. Collectively, these miRNA-target mRNA pairs may contribute to the defense response against *S. sclerotiorum*.

In the comparison group *ago2*/WT, 137 reversely expressed miRNA-target mRNA pairs involving 24 miRNAs were identified (Supplementary Table S13). The functions of these target genes were diversely involved in a variety of cellular and molecular processes, including defense, development, metabolism, transcription, signal transduction and redox homeostasis, which indicated that loss of AGO2 may affect many aspects of the plants. Among them, 9 miRNA-target mRNA pairs were obviously related with defense (Supplementary Table S15). These included ath-miR391-3p and targets *GSTU2* and *JRL22* and ath-miR393a-3p and targets *GSTU2*, *MBP2* and *WAK1*. These results showed that AGO2 may affect the expression of many defense-related genes by miRNAs so as to influence the resistance of Arabidopsis to *S. sclerotiorum*.

Moreover, 490 reversely expressed DEM-DEG pairs involving 29 DEMs and 249 DEGs in comparison of *ago2* with WT after inoculation (*ago2-Ss*/WT-*Ss*) were identified (Supplementary Table S13). The DEM-DEG pairs that might be related to resistance were listed in Table 2. These pairs involved 14 DEMs and 22 DEGs. In some pairs, several defense-related DEGs were predicted to be targeted by one DEM. Among the important target DEGs were 5 resistance genes targeted by 5 different miRNAs, including two CC-NBS-LRR type R genes targeted by ath-miR158a-5p and ath-miR865-3p, respectively, a LRR family protein by ath-miR1888a, RMG1 by 2_13869-5p and a TIR-NBS-LRR type R gene by 2_13869-5p (Table 2). Other interesting targets included some redox homeostasis related genes such as *RbohF* and some *GST* genes. Collectively, these miRNA-target gene pairs may play a role in AGO2-mediated resistance to *S. sclerotiorum*.

**TABLE 2:** Potential AGO2-regulated DEM-DEG pairs involved in the defense of Arabidopsis against *S. sclerotiorum.*

DEM	Fold change (*p-*Value)	Regulation (*ago2-Ss/*WT-*Ss*)	Predicted DEG	Annotation	Fold change (*q*-Value)	Regulation (*ago2-Ss/*WT-*Ss*)
ath-miR158a-5p	−1.63 (0)	Down	AT1G64195	Defensin-like protein 35	2.13 (0)	Up
			AT1G50180	CC-NBS-LRR	2.02 (0.08)	Up
			AT5G49520	WRKY transcription factor 48	2 (0)	Up
ath-miR170-5p	−2.19 (0.001)	Down	AT1G17750	PEPR2	3.01 (0)	Up
			AT1G21245	Wall-associated receptor kinase 3	2.08 (0)	Up
ath-miR391-3p	−5.68 (0)	Down	AT1G17750	PEPR2	3.01 (0)	Up
			AT2G29450	Glutathione S-transferase U5	2.98 (0)	Up
			AT2G29480	Glutathione S-transferase U2	2.4 (0)	Up
			AT3G13650	Dirigent protein 7	2.11 (0)	Up
ath-miR393b-3p	−1.82 (0)	Down	AT2G29480	Glutathione S-transferase U2	2.4 (0)	Up
ath-miR472-5p	−1.81 (0.003)	Down	AT5G01900	WRKY transcription factor 62	3.54 (0)	Up
			AT3G12500	Basic endochitinase B	2.53 (0.009)	Up
			AT1G64060	Respiratory burst oxidase homolog protein F	2.25 (0)	Up
ath-miR822-3p	−1.85 (0.001)	Down	AT1G01480	1-aminocyclopropane-1-carboxylate synthase 2	2.49 (0)	Up
			AT1G32640	Transcription factor MYC2	2.02 (0)	Up
ath-miR850	−2.44 (0)	Down	AT2G35000	E3 ubiquitin-protein ligase ATL9	2.19 (0.015)	Up
ath-miR8175	1.52 (0.008)	Up	AT2G28670	Dirigent protein 10	−2.01 (0.024)	Down
ath-miR861-3p	−1.59 (0.025)	Down	AT1G17750	PEPR2	3.01 (0)	Up
			AT1G01480	1-aminocyclopropane-1-carboxylate synthase 2	2.49 (0)	Up
ath-miR865-3p	−1.74 (0.039)	Down	AT1G50180	CC-NBS-LRR	2.02 (0.008)	Up
ath-miR1888a	−1.93 (0.027)	Down	AT2G34930	Disease resistance family protein/LRR family protein	2.89 (0)	Up
			AT2G17480	MLO-like protein 8	2.18 (0)	Up
1_47029-5p	−1.68 (0.007)	Down	AT1G19570	Glutathione S-transferase DHAR1	2.06 (0)	Up
2_13869-5p	−1.65 (0.027)	Down	AT5G41740	Disease resistance protein (TIR-NBS-LRR) class)	2.05 (0)	Up
			AT4G11170	Disease resistance protein At4g11170 (RMG1)	2.01 (0)	Up
4_5020-5p	−2.98 (0.021)	Down	AT1G17380	Protein TIFY 11A	2.08 (0)	Up

The most attractive DEM-DEG pairs to us are ATSR pairs. DEMs of these pairs were expressed significantly differentially in both WT-*Ss*/WT and *ago2-Ss*/*ago2* comparisons but to the opposite direction while DEGs of these pairs should be reversely expressed to the corresponding DEMs and expressed significantly differentially and reversely in WT-*Ss*/WT and *ago2-Ss*/*ago2* comparisons or only significantly differentially expressed in one of the comparisons. The potential ATSR pairs were listed in Table 3. Among them were DEM-DEG pairs ath-miR398b-5p/ath-miR398c-5p-RLP3/GLR3.6/GH3.17/EXPA8, ath-miR398b-3p/ath-miR398c-3p-NIMIN1/CSD1/CCS and ath-miR408-3p/LAC13. The DEGs of these pairs are known to play important roles in disease resistance or susceptibility (references in Table 3). Therefore, these DEM-DEG pairs represent promising ATSR pairs participating in resistance or susceptibility regulation.

**TABLE 3:** Potential ATSR DEM-DEG pairs.

miRNA ID	Fold Change (*p*-Value)		Target gene ID	Fold Change (*q*-Value)*		Description	Function
	WT-*Ss*/WT	*ago2-Ss/ago2*		WT-*Ss*/WT	*ago2-Ss/ago2*		
ath-miR397b	0.21 (0.001)	3.35 (0.011)	AT5G52910	−	0.45 (0.005)	Timeless family protein ATIM	Regulation of circadian rhythm
			AT3G22810	−	0.47 (0)	FORKED-LIKE family member	Auxin-activated signaling
ath-miR398b-3p/ath-miR398c-3p	0.36 (0)	3.67 (0)	AT1G02450	4.23 (0.002)	−	NIMIN1 (NIM1-Interacting 1)	SA signaling and PR gene expression
			AT1G08830	2.49 (0)	1.23 (0.05)	CSD1 (Superoxide dismutase [Cu-Zn] (1)	Removal of superoxide radicals
			AT1G12520	−	0.4 (0)	CCS (Copper chaperone for superoxide dismutase)	Delivery of Cu to CSD
ath-miR398b-5p/ath-miR398c-5p	0.31 (0)	2.66 (0)	AT1G17250	3.43 (0.001)	−	RLP3 (receptor like protein 3)/RFO2 (Resistance to *Fusarium oxysporum* 2)	Disease resistance
			AT1G28130	−	0.36 (0.002)	Indole-3-acetic acid-amido synthetase GH3.17	Disease development
			AT2G40610	−	0.39 (0)	EXPA8 (Expansin-A8)	Cell wall loosening and organization
			AT3G51480	−	0.39 (0)	GLR3.6 (Glutamate receptor 3.6)	Calcium flux, disease resistance
ath-miR408-3p	0.49 (0)	2.44 (0)	AT5G14610	−	0.50 (0)	DEAD-box ATP-dependent RNA helicase 46	RNA-binding
			AT5G07130	−	0.44 (0)	Laccase-13	Oxidative stress, lignification

*“−”indicates the difference is not significant.

### Quantitative RT-PCR Validation of Significant DE miRNAs and mRNAs

**FIGURE 3:**
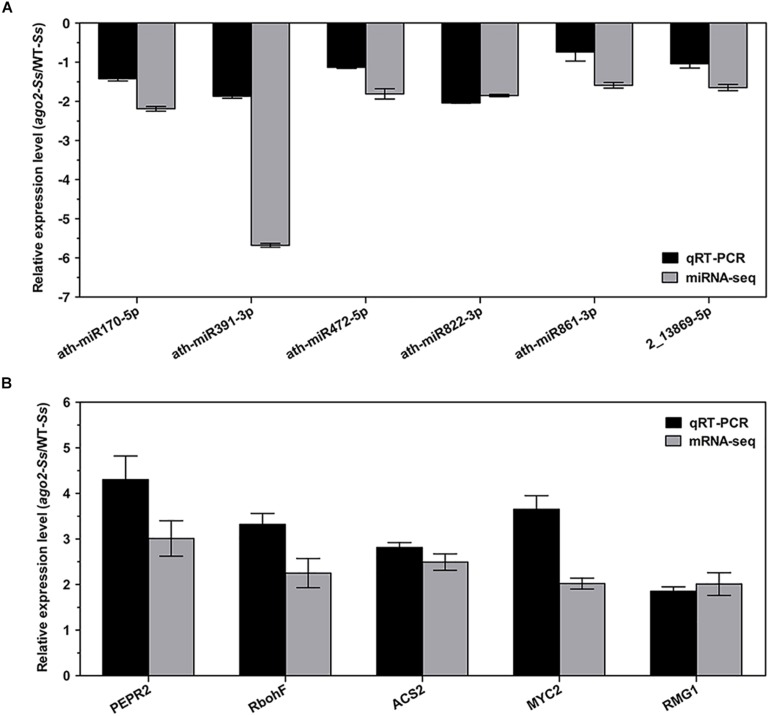
Experimental confirmations of RNA-seq data on differential expression of the selected 6 DEM-DEG pairs of the comparison *ago2-Ss*/WT*-Ss* by qRT-PCR analyses. qRT-PCR results on relative expression of the selected DEMs **(A)** and DEGs **(B)** in *S. sclerotiorum*-inoculated *ago2-1* mutant (*ago2-Ss*) to *S. sclerotiorum*-inoculated wild-type (WT*-Ss*) were shown in parallel with the RNA-seq results. RT-PCR data represent the mean ± SE of three independent experiments.

Although the DEMs and DEGs identified from the above integrated analyses should have verified each other in principle, in order to further validate the RNA-seq and sRNA-seq data, 6 DEMs and 5 DEGs of the comparison group *ago2-Ss*/WT-*Ss*, which may play a role in AGO2-mediated plant defense response, were selected for expression profile analyses by qRT-PCR. These 5 DEGs were manually selected as representatives for their potential roles in plant defense according to their annotations and their potential relationship with *S. sclerotiorum*-responsive miRNAs. These genes included *PEPR2* (ath-miR170-5p, ath-miR391-3p, ath-miR861-3p), *RbohF* (ath-miR472-5p), *MYC2* (ath-miR822-3p), *ACS2* (ath-miR822-3p, ath-miR861-3p) and *RMG1* (2_13869-5p). The results of RT-qPCR revealed that most of these mRNAs/miRNAs shared similar expression tendency with those from RNA-seq/sRNA-seq data (Figure 3 and Table 2). Although there were some quantitative differences between the two analytical platforms, the similarities between these two methods suggested that the RNA-seq/sRNA-seq data are reproducible and reliable.

### Data-Mining for AGO2-Binding Abundance and *Pseudomonas syringae*-Responsiveness of DEMs Identified in This Study

Arabidopsis AGO2-associated sRNA population after *Pst* (avrRpt2) and mock inoculations was identified through Illumina deep sequencing using a transgenic AGO2:3HA-AGO2 line (Zhang et al., 2011). In order to know whether the 46 DEMs identified in comparison groups *ago2*/WT and *ago2-Ss*/WT*-Ss* in this study could be bound by AGO2 directly, we searched these DEMs in their AGO1- and AGO2-immunoprecipitated (IP) sequencing data set deposited in NCBI (GSE26161). Searching results showed that among the 46 DEMs, 32 could be loaded into AGO2 constitutively and the AGO2-binding abundance of 35 DEMs was enhanced by *Pst*-infection (Table 4). Among the most abundant AGO2-bound sRNAs are ath-miR391-3p, ath-miR393b-3p and ath-miR472-5p. They had the 5′ terminal A, a feature of AGO2-associated sRNAs, and accumulated with more than 10000 reads per million genome-matched sequences (mgs). Their binding with AGO2 might be enhanced by *Pst*-infection (Table 4). Interestingly, these three DEMs were down-regulated in *ago2* compared with WT plants after *S. sclerotiorum* inoculation (*ago2-Ss*/WT-*Ss*). Together, these results indicated that these three DEMs might play an important role in plant immunity against both bacterial and fungal pathogens in an AGO2-dependent manner. Further comparison of the enrichment of the DEMs in AGO2-IP and AGO1-IP fractions showed that 21 DEMs were only or dominantly existed in AGO2-IP fraction and thus were likely to be specifically bound by AGO2 but not AGO1, while 7 DEMs appeared similarly in both AGO2-IP and AGO1-IP fractions and thus might be bound by both AGO1 and AGO2 (Table 4). Taken together, the DEMs identified in comparison groups *ago2*/WT and/or *ago2-Ss*/WT*-Ss* in this study were likely to be regulated by AGO2 and most of them could be bound by AGO2 directly.

**TABLE 4:** AGO2-binding abundance and *Pseudomonas syringae*-responsiveness of DEMs identified in this study.

miRNA	Sequence	DE in this study		Constitutive AGO2-binding	*Pst*-responsive AGO2-binding	Constitutive AGO1-binding	*Pst*-responsive AGO1-binding
		*ago2*/WT	*ago2-Ss*/WT-*Ss*				
ath-miR158a-5p	ctttgtctacaattttggaaa		Down	30	64	25	
ath-miR160c-3p	cgtacaaggagtcaagcatga		Up				
ath-miR164a	tggagaagcagggcacgtgca		Up	11	64	1481	1861
ath-miR164b-5p	tggagaagcagggcacgtgca		Up	11	64	1481	1861
ath-miR168a-3p	cccgccttgcatcaactgaat	Up		3	1		
ath-miR169g-3p	tccggcaagttgaccttggct	Up					
ath-miR170-5p	tattggcctggttcactcaga		Down	4449	13909	992	706
ath-miR171a-5p	tattggcctggttcactcaga		Down	4449	13909	992	706
ath-miR171b-5p	agatattagtgcggttcaatc	Down		181	613	1	1
ath-miR1888a	taagttaagatttgtgaagaa		Down	112	552	1409	1390
ath-miR391-3p	acggtatctctcctacgtagc	Down	Down	3497	14030	91	134
ath-miR391-5p	ttcgcaggagagatagcgcca	Up			5	151	290
ath-miR393a-3p	atcatgctatctctttggatt	Down		37	370		40
ath-miR393b-3p	atcatgcgatctctttggatt	Down	Down	17732	69558		57
ath-miR395b	ctgaagtgtttggggggactc	Down		20	102	734	692
ath-miR395c	ctgaagtgtttggggggactc	Down		20	102	734	692
ath-miR395f	ctgaagtgtttggggggactc	Down		20	102	734	692
ath-miR397b	tcattgagtgcatcgttgatg	Down	Up	6	49	16	24
ath-miR398a-3p	tgtgttctcaggtcacccctt	Down	Up				66
ath-miR398b-3p	tgtgttctcaggtcacccctg	Down	Up		2	58	172
ath-miR398b-5p	agggttgatatgagaacacac	Down	Up	844	4373	1	
ath-miR398c-3p	tgtgttctcaggtcacccctg	Down	Up		2	58	172
ath-miR398c-5p	agggttgatatgagaacacac	Down	Up	844	4373	1	
ath-miR408-3p	atgcactgcctcttccctggc	Down		2740	12566	365	202
ath-miR408-5p	acagggaacaagcagagcatg		Up	403	2074	818	2091
ath-miR472-5p	atggtcgaagtaggcaaaatc	Down	Down	3395	19153	86	79
ath-miR5026	actcataagatcgtgacacgt	Down		10450	45060	137	648
ath-miR5654-5p	ataaatcccaacatcttcca	Down		7	49		
ath-miR5663-5p	agctaaggatttgcattctca		Up	25	123		
ath-miR8175	gatccccggcaacggcgcca		Up		8		
ath-miR822-3p	tgtgcaaatgctttctacagg		Down	121	725	17	
ath-miR840-5p	acactgaaggacctaaactaac		Down	945	4304		
ath-miR841b-5p	tacgagccactggaaactgaa	Down		1	8		
ath-miR843	tttaggtcgagcttcattgga	Down		33	176	2318	833
ath-miR846-3p	ttgaattgaagtgcttgaatt		Up	4	9	71	1
ath-miR850	taagatccggactacaacaaag	Down	Down	105	812	407	586
ath-miR861-3p	gatggatatgtcttcaaggac		Down				
ath-miR865-3p	tttttcctcaaatttatccaa		Down				
1_41269-5p	tccgatgtcgtccagcggttaggata	Down		2	111	59	
1_47029-3p	attgcatatctcaggagcttt		Down		16		
1_47029-5p	agctgctaagctatggatccc		Down		2		
2_13869-5p	atcagctcggatgactctgtcagc		Down	4	4		
4_5020-5p	tttgttttgttttgattggttca		Down				
4_6055-5p	atcagctcggatgactctgtcagc		Down	4	4		
5_31160-3p	gagaatgatgaaccaattagatg		Up				
Pt_1172-3p	gaggaaataaaagatctt		Down				

### Resistance of *gstu2*, *gstu5*, and *rbohf* Mutant Plants to *S. sclerotiorum*

**FIGURE 4:**
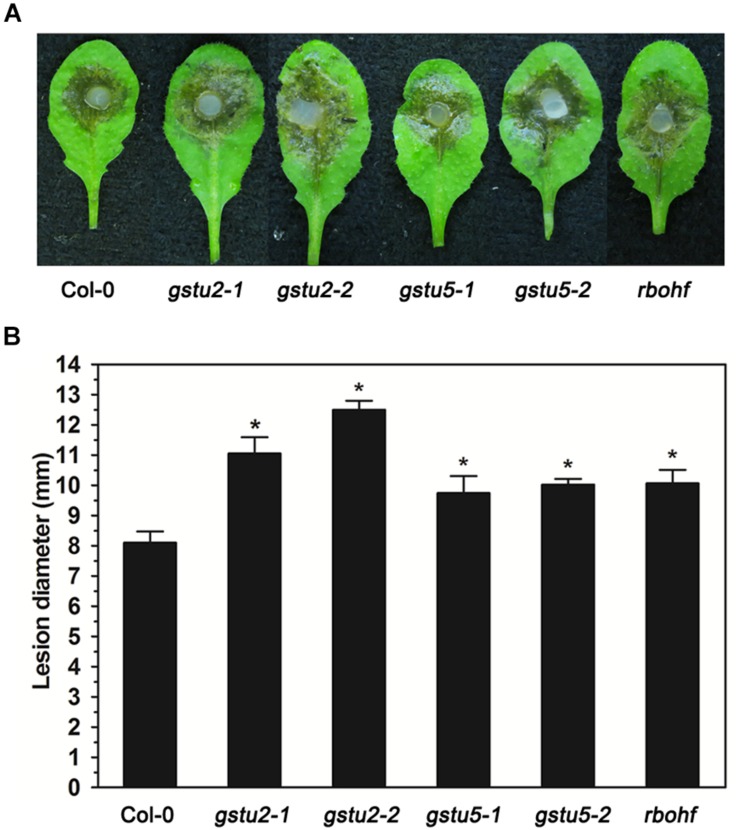
Resistance to *S. sclerotiorum* was reduced in Arabidopsis *gstu2*, *gstu5* and *rbohf* mutants. **(A)** Disease symptoms of the *gstu2*, *gstu5* and *rbohf* mutant plants and Col-0 wild-type plants after inoculation with *S. sclerotiorum*. Photographs were taken at 28 hpi. **(B)** Statistical analysis of disease severity. The inoculation analysis was performed three times, each in at least 10 plants for all mutants. The experiments were conducted three times, each containing three replicates for all genes. The expression data were statistically analyzed using SPSS software. Significance of the differences between mean values was determined with Student’s *t*-test. Error bars represent SD, while asterisks (*) indicate significant difference at *p-*Value < 0.05.

Target genes and miRNAs listed in Tables 2, 3 may be both regulated by AGO2 and involved in the interaction between Arabidopsis and *S. sclerotiorum*. Among the lists were a variety of genes related with redox homeostasis. In order to verify the function of these target genes in the resistance to *S. sclerotiorum*, we chose three of them (*GSTU2*, *GSTU5*, and *RbohF*) to test the sensibility of their corresponding Arabidopsis mutant plants to *S. sclerotiorum*. As shown in Figure 4, comparing to WT plants, all these three mutant plants exhibited larger lesion area and more severe necrosis, which indicated that these mutant plants were more susceptible to *S. sclerotiorum* (Figure 4). These data demonstrated that *GSTU2*, *GSTU5* and *RbohF* play a positive role in regulating the resistance of Arabidopsis to *S. sclerotiorum*, and implied that the identified miRNA-target gene pairs in this study might indeed contribute to the AGO2-mediated or AGO2-independent defense response against *S. sclerotiorum* according to the comparison groups.

## Discussion

Our previous work indicated that RNA silencing played a vital role in the interaction between plant and *S. sclerotiorum* (Cao et al., 2016a, b). AGO2, a key component in RNA silencing pathway, significantly affects the resistance to *S. sclerotiorum* (Cao et al., 2016b). In this study, we performed correlative analyses for both miRNAome and transcriptome of Arabidopsis plants of WT and *ago2-1* mutant constitutively and in response to *S. sclerotiorum*. Consequently, we identified AGO2-associated miRNAs and defense responses against *S. sclerotiorum* at the genome-wide level. As far as we know, this is the first report of AGO2-associated miRNAs related to antifungal defense. Our results establish a link between AGO2-dependent RNA silencing and resistance against a necrotrophic fungal pathogen.

Correlative analysis for miRNAome and transcriptome owns obvious advantages over separately analysis of them. Firstly, correlative analyses can effectively identify functional miRNAs and their target genes simultaneously by simply searching for the miRNA-target gene pairs with reverse expression tendency under the same treatment and conditions based on the recognition that in most cases, miRNAs negatively regulate the expression of their target mRNAs by degrading target RNAs. Since the correlative analysis is conducted for DEMs and DEGs, therefore the identified miRNAs and target genes are both differentially expressed, which increases the possibility that these identified miRNA-target gene pairs are indeed importantly involved in the biological process of interest. In addition, correlative analyses lead to genome-wide identification of functional miRNA-target gene pairs, and thus can comprehensively reveal miRNA-mediated regulation mechanism. In this study, integrated miRNAome and transcriptome analysis employing *ago2* mutant resulted in identification of 622, 137, 490, and 1091 DEM-predicted DEG pairs involving 29, 24, 29, and 26 DEMs of the comparison *WT-Ss*/WT, *ago2*/WT, *ago2-Ss*/WT*-Ss* and *ago2-Ss*/*ago2*, respectively. Following the DEM-DEG pair prediction results, using Arabidopsis mutants, we further demonstrated the essential role of *GSTU2*, *GSTU5* and *RBOHF* in the AGO2-mediated *S. sclerotiorum* resistance. This represents the first genetic evidence on contribution of *GST* genes to plant resistance against *S. sclerotiorum*. The miRNA–target gene pairs identified in this study provide good candidates to further elucidate the AGO2-dependent susceptibility to and resistance against *S. sclerotiorum*.

The correlative analysis data are reliable and the DEMs and DEGs identified in this study are functionally related considering the following facts. Firstly, the correlative analysis was performed for the DEMs and DEGs, and thus the significance of differential expression of the DEMs and DEGs in the predicted DEM-DEG pairs have been verified each other. Secondly, the reverse differential expression of the DEMs and DEGs in the predicted DEM-DEG pairs had been experimentally confirmed as exemplified by the selected 6 DEM-DEG pairs (Figure 3). Thirdly, the AGO2-binding abundance and pathogen-responsiveness of the DEMs have been verified by data-mining of the AGO2-IP sequencing data reported previously (Zhang et al., 2011). Finally, some DEGs and DEMs have been previously reported to play an important role in defense against *S. sclerotiorum* and/or other pathogens. For instance, ath-miR393b-3p, also named ath-miR393b^∗^, is the only defense-related miRNA that has been experimentally confirmed to be functionally associated with AGO2 to date. It targets *MEMB12* for silencing to promote the exocytosis of antimicrobial PR proteins thereby contributing to immunity against the bacterial pathogen *Pst* (Zhang et al., 2011). Intriguingly, in this study, we found that this miRNA is also significantly down-regulated in *ago2* mutant compared with WT both constitutively (*ago2*/WT) and in response to *S. sclerotiorum* (*ago2-Ss*/WT*-Ss*). Additionally, many DEGs identified in this study were reported to contribute to defense against *S. sclerotiorum* and/or other pathogens. Among these DEGs is *PAD3*, a key gene in camalexin synthesis pathway and defense against *S. sclerotiorum* (Stotz et al., 2011). *PAD3* was up-regulated by 1136.5 folds after *S. sclerotiorum* inoculation (Supplementary Table S5). Other DEGs known to be involved in defense against *S. sclerotiorum* include WRKY33 and many ROS and hormone regulatory genes. Besides, a set of PTI and DTI related genes including *BAK1*, *LYK5*, *WAK1*, *Peps*, and *PEPR2*, important defense signaling genes *EDS5* and *NDR1*, were also among the list of our DEGs (Supplementary Table S3).

It is noticeable that some DEMs altered differently in the performed four comparisons. For example, miRNAs ath-miR397b, ath-miR398b-3p/ath-miR398c-3p, and ath-miR398b-5p/ath-miR398c-5p were down-regulated in *ago2-1* mutant compared to WT (*ago2*/WT) and in WT plants after *S. sclerotiorum* inoculation (WT-*Ss*/WT), but up-regulated in *S. sclerotiorum*-inoculated *ago2-1* mutant compared to *S. sclerotiorum*-inoculated WT (*ago2-Ss*/WT-*Ss*) and group *ago2-Ss/ago2* (Supplementary Table S12). These data indicated that mechanisms underlying the effect of AGO2 and the pathogen *S. sclerotiorum* on the biogenesis and accumulation of these miRNAs varies significantly.

It is also the case for the reversely expressed DEM-DEG pairs (Supplementary Table S13). Majority (440/490) of the DEM-DEG pairs identified from the comparison *ago2-Ss*/WT-*Ss* were differentially expressed exclusively in this comparison, rather not in other three comparisons (Figure 2C and Supplementary Table S13). Considering that DEMs were significantly overlapped between the four comparisons, the lack of overlapping DEM-DEG pairs between *ago2-Ss*/WT-*Ss* and the other two comparisons implied that the target DEGs of the DEMs differs obviously between response to *S. sclerotiorum* and mutation of *AGO2*. In addition, in some pairs, several defense-related genes were predicted for one miRNA. Among these pairs were ath-miR158a-5p and its predicted target genes encoding defensin-like protein 35, CC-NBS-LRR proteins and WRKY48, ath-miR170-5p and its predicted target genes encoding PEPR2 and WAK3, and ath-miR391-3p and its predicted target genes encoding PEPR2, GSTU2/U5 and DIR7. It is noticeable that all these miRNA-target mRNA pairs expect ath-miR8175 and its target gene *DIR10* showed the same expression pattern in which the miRNA was down-regulated while the corresponding target genes were all up-regulated in the comparison *ago2-Ss*/WT*-Ss* (Table 2). This may represent the effective way for miRNA to regulate a variety of mRNA targets synergistically for the same biological process.

As a critical component of the RNAi pathway, AGO2 might regulate plant immunity and susceptibility through the action of its bound sRNAs. Therefore, we conducted a genome-wide integrated analysis of miRNAome and transcriptome following *S. sclerotiorum* infection in both *ago2-1* mutant and WT plants to gain an insight into the AGO2-dependent regulation of susceptibility and resistance to *S. sclerotiorum*. By this analytic strategy, we identified some candidate miRNA-mRNA pairs involved in the interaction between Arabidopsis and *S. sclerotiorum* or/and regulated by AGO2 (Table 2 and Supplementary Tables S14, S15). Among them, over 40 miRNA-mRNA pairs might be involved in AGO2-mediated defense against *S. sclerotiorum* (Tables 2, 3). The interesting mRNAs include three *GST* genes (*GSTU2*, *GSTU5* and *DHAR1*) and *RbohF*. GSTs play essential role in detoxification and abiotic stress response. However, their contribution to resistance against pathogens and especially their functional mechanisms are less understood (Dixon et al., 2010; Gullner et al., 2018). We found that *gstu2* and *gstu5* mutant plants were more sensitive to *S. sclerotiorum* than WT plants (Figure 4), which indicated that these two genes might positive regulate the resistance to *S. sclerotiorum*. *RbohF* is one of the ten RBOHs genes present in Arabidopsis. It has a limited effect on ROS production compared with *RbohD*, but exhibits a much greater effect on cell death (Torres et al., 2002). ROS and cell death are both key factors during the interaction between plants and *S. sclerotiorum*. It was reported that *Atrbohd/Atrbohf* double mutant plants showed an extremely susceptible phenotype, which demonstrated the above conclusion (Perchepied et al., 2010). In the present work, inoculation analysis of *rbohf* mutant plants suggested that *RbohF* might positively regulate the resistance to *S. sclerotiorum* (Figure 4). Based on the results of integrated analysis of sRNA-seq and RNA-seq, these DEGs are regulated by DEMs ath-miR391-3p, ath-miR393b-3p and ath-miR472-5p, respectively (Table 2). These miRNAs were reported to be AGO2-bound (Table 4). Collectively, we supposed that AGO2 binds ath-miR391-3p, ath-miR393b-3p and ath-miR472-5p to repress the expression of these *GST* and *RbohF* genes. In response to *S. sclerotiorum* infection, plant adjusts the loading of these miRNAs to AGO2, thereby modifies expression of *GST* and *RbohF* genes so as to affect the redox homeostasis and cell death status, which finally changed the resistance of Arabidopsis to *S. sclerotiorum.* Similar regulatory mechanisms may also be applied to the other DEM-DEG pairs listed in Tables 2, 3 and Supplementary Tables S14, S15, including the interesting DEGs *PEPR2*, a receptor of DAMPs Peps, RLP3, NIMIN1, GLR3.6, two WRKY genes *WRKY48* and *WRKY62*, hormone signaling related genes *ACS2* and *MYC2*. Taking together, AGO2 might affect the resistance of Arabidopsis to *S. sclerotiorum* by regulating the expression of these DEM-DEG pairs.

**FIGURE 5:**
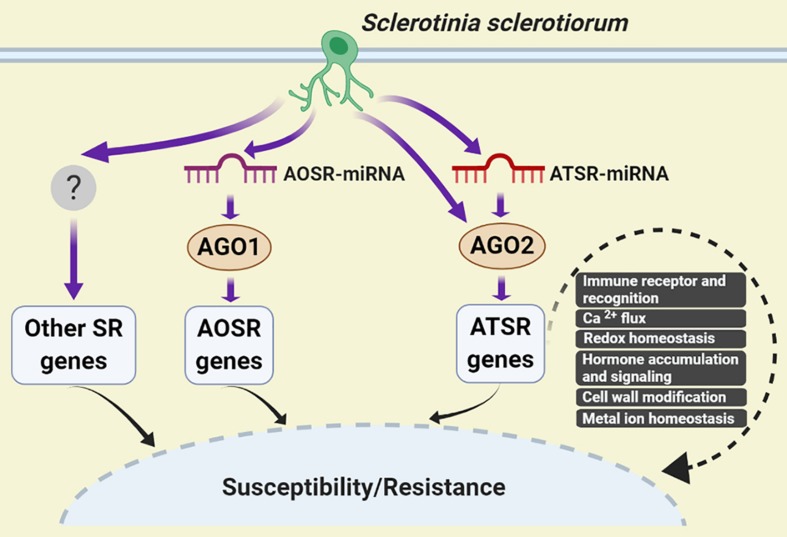
A working model for AGO2-mediated regulation of Arabidopsis–*S. sclerotiorum* interactions. *S. sclerotiorum*-responsive genes-mediated regulation of plant susceptibility and resistance could be dependent and independent of AGOs. The former mainly comprised AGO1- and AGO2-dependent pathways. In respond to *S. sclerotiorum* infection, accumulation level of ATSR-miRNAs altered rapidly, which caused the change of ATSR gene transcript abundance in the reverse trend to the corresponding ATSR-miRNAs via function of ATSR-miRNA-AGO2 RISC complex. This led to modulation of immune recognition, calcium flux, redox homeostasis, hormone accumulation and signaling, cell wall modification and metal ion homeostasis, thereby regulated the plant susceptibility and/or resistance to *S. sclerotiorum*. Some ATSR genes might be regulated by AGO2 independent of miRNA. AOSR-miRNA/AGO1-dependent regulation of AOSR gene transcript accumulation function in parallel. AOSR, AGO1-dependent *S. sclerotiorum* -responsive; ATSR, AGO2-dependent *S. sclerotiorum* -responsive; SR, *S. sclerotiorum*-responsive.

Based on our results, a working model for AGO2-mediated regulation of Arabidopsis-*S. sclerotiorum* interactions was proposed. Upon *S. sclerotiorum* infection, accumulation level of ATSR-miRNAs such as ath-miR398b-5p/ath-miR398c-5p, ath-miR398b-3p/ath-miR398c-3p and ath-miR408-3p, altered rapidly, which were loaded to AGO2 and assembled into RISC complex, and caused the change of abundance of ATSR gene transcripts to the opposite direction to the corresponding ATSR-miRNAs, which led to modulation of immune recognition (RLP3, PEPR2, MD2-like, TRAF-like), calcium flux (GLR3.6), redox homeostasis (GSTs, RBOHF, CSD1, FSD1, CCS, and LAC13), hormone accumulation and signaling (GH3.17, NIT2, ACS2, MYC2, and NIMIN1), cell wall modification (EXPA8) and metal ion homeostasis (FEP2/IMA2), thereby regulated the plant susceptibility and/or resistance to the necrotrophic pathogen *S. sclerotiorum*. Some ATSR genes might be regulated by AGO2 independent of miRNA. A similar AOSR (AGO1-dependent *S. sclerotiorum*-responsive)-miRNA/AGO1-dependent regulation of AOSR gene transcript accumulation should also exist (Figure 5).

The future work includes further experimental verification of the predicted target DEGs of the DEMs by analyses such as 5′ RNA ligase mediated rapid amplification of cDNA ends (5′RLM RACE). Effect of *S. sclerotiorum* infection on the AGO2-DEMs binding is also worthy of further exploring. The function mechanism of the ATSR DEM-DEG pairs in the resistance against the devastating pathogen *S. sclerotiorum* will be the important aspect of the following work. Additionally, it should be noted that AGO2-mediated gene silencing is implemented via not only target gene degradation but also translational repression (Fatyol et al., 2016). In the present study, we only focused on the genes that are regulated by AGO2 at post-transcriptional level by correlation analyses. Therefore, we cannot rule out the possibility that those genes, predicted to be targets of DEMs but did not show significantly differential expression in comparison groups *ago2*/WT and *ago2-Ss*/WT*-Ss*, might be regulated by AGO2 in a translational repression mechanism. Therefore, it is also interesting to identify this type of AGO2-regulated genes.

Additionally, AGO-mediated gene silencing mechanisms also function in *S. sclerotiorum* (Neupane et al., 2019) and bidirectional cross-kingdoms RNAi occurs in plant-necrotrophic pathogens interaction systems (Wang M. et al., 2016). Whether plant AGO2 affects *S. sclerotiorum* by directly modulating pathogen gene silencing awaits confirmation.

## Data Availability Statement

The datasets generated for this study can be found in the NCBI SRA database under the accession numbers PRJNA607858 and PRJNA607657.

## Author Contributions

X-ZC coordinated the project, conceived the study, and participated in its design and coordination. J-YC and Y-PX analyzed the RNA-Seq data and performed the experiments. X-ZC and J-YC prepared the manuscript. All authors read and approved the final manuscript.

## Conflict of Interest

The authors declare that the research was conducted in the absence of any commercial or financial relationships that could be construed as a potential conflict of interest.

## Footnotes

1 https://david.ncifcrf.gov

2 http://www.ncbi.nlm.nih.gov/

3 http://www.mirbase.org/

4 http://sourceforge.net/projects/mireap/

## Abbreviations

AGOs: Argonautes
AOSR: AGO1-dependent *Sclerotinia sclerotiorum*-responsive
ATSR: AGO2-dependent *Sclerotinia sclerotiorum*-responsive
DAMP: damage associated molecular pattern
DEGs: differentially expressed genes
DEMs: differentially expressed miRNAs
diRNAs: DSB-induced small RNAs
DIRs: Dirigent proteins
DSB: DNA double strand breaks
ET: ethylene
FPKM: Fragments per kilobase mapped
JA: jasmonic acid
JRLs: jacalin-related lectins
NCBI: National Center for Biotechnology Information
NO: nitric oxide
PAMP: pathogen associated molecular pattern
Peps: plant elicitor peptides
PR protein: pathogenesis-related protein
ROS: reactive oxygen species
RH: relative humidity
RT-qPCR: reverse transcription quantitative polymerase chain reaction
RBOHs: respiratory burst oxidase homologues
SR: *Sclerotinia sclerotiorum*-responsive
sRNA: small RNA
vsiRNAs: virus-derived small interfering RNAs
vasiRNAs: virus-activated siRNAs
WT: wild-type.
