# Supplementary material for: Integrated miRNAome and Transcriptome Analysis Reveals Argonaute 2-Mediated Defense Responses Against the Devastating Phytopathogen Sclerotinia sclerotiorum
Source: Front Plant Sci. 2020 Apr 29;11:500. doi: 10.3389/fpls.2020.00500 (PMC7201365; doi:10.3389/fpls.2020.00500)
Supplement: Supplementary file 1 [file Data_Sheet_1.PDF]

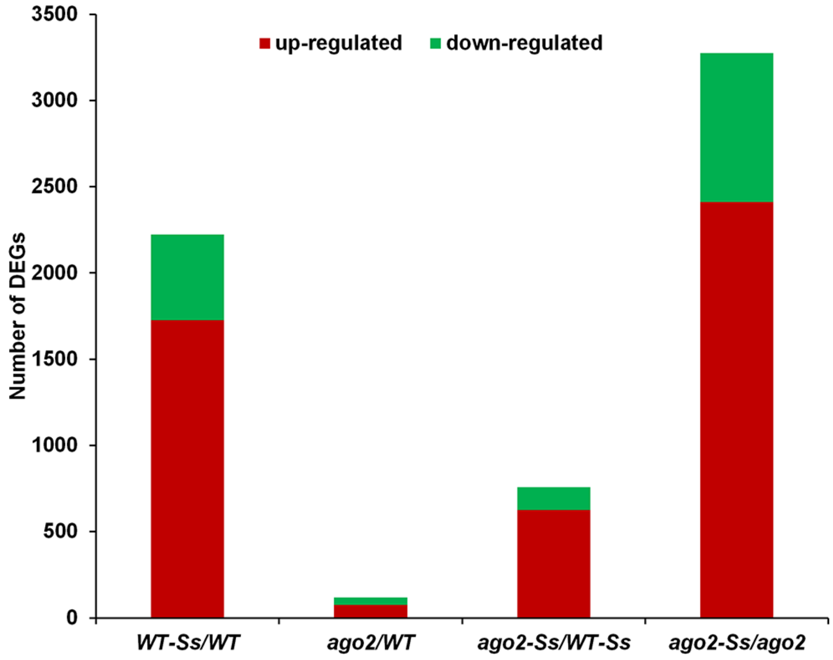

Figure S1. Distribution of differentially expressed genes from each comparison group. WT-*Ss*/WT: comparison of genes differentially expressed between mock and *S. sclerotiorum*-infected WT plants; *ago2*/WT: comparison of genes differentially expressed between *ago2* and WT plants in the absence of the pathogen; *ago2-Ss/*WT-*Ss*: comparison of genes differentially expressed between *S. sclerotiorum* infected *ago2* and WT plants; *ago2-Ss/ago2*: comparison of genes differentially expressed between *S. sclerotiorum* infected *ago2* and *ago2* plants.
